# Potassium and Ionic Strength Effects on the Conformational and Thermal Stability of Two Aldehyde Dehydrogenases Reveal Structural and Functional Roles of K^+^-Binding Sites

**DOI:** 10.1371/journal.pone.0054899

**Published:** 2013-01-24

**Authors:** Georgina Garza-Ramos, Carlos Mújica-Jiménez, Rosario A. Muñoz-Clares

**Affiliations:** 1 Departamento de Bioquímica, Facultad de Medicina, Universidad Nacional Autónoma de México, México, Distrito Federal, México; 2 Departamento de Bioquímica, Facultad de Química, Universidad Nacional Autónoma de México, México, Distrito Federal, México; National Institute for Medical Research, Medical Research Council, United Kingdom

## Abstract

Many aldehyde dehydrogenases (ALDHs) have potential potassium-binding sites of as yet unknown structural or functional roles. To explore possible K^+^-specific effects, we performed comparative structural studies on the tetrameric betaine aldehyde dehydrogenase from *Pseudomonas aeruginosa* (PaBADH) and on the dimeric BADH from spinach (SoBADH), whose activities are K^+^-dependent and K^+^-independent, respectively, although both enzymes contain potassium-binding sites. Size exclusion chromatography, dynamic light scattering, far- and near-UV circular dichroism, and extrinsic fluorescence results indicated that in the absence of K^+^ ions and at very low ionic strength, PaBADH remained tetrameric but its tertiary structure was significantly altered, accounting for its inactivation, whereas SoBADH formed tetramers that maintained the native tertiary structure. The recovery of PaBADH native tertiary-structure was hyperbolically dependent on KCl concentration, indicating potassium-specific structuring effects probably arising from binding to a central-cavity site present in PaBADH but not in SoBADH. K^+^ ions stabilized the native structure of both enzymes against thermal denaturation more than did tetraethylammonium (TEA^+^) ions. This indicated specific effects of potassium on both enzymes, particularly on PaBADH whose apparent *T*_m_ values showed hyperbolical dependence on potassium concentration, similar to that observed with the tertiary structure changes. Interestingly, we also found that thermal denaturation of both enzymes performed in low ionic-strength buffers led to formation of heat-resistant, inactive soluble aggregates that retain 80% secondary structure, have increased β-sheet content and bind thioflavin T. These structured aggregates underwent further thermal-induced aggregation and precipitation when the concentrations of KCl or TEACl were raised. Given that PaBADH and SoBADH belong to different ALDH families and differ not only in amino acid composition but also in association state and surface electrostatic potential, the formation of this kind of β-sheet pre-fibrillar aggregates, not described before for any ALDH enzyme, appear to be a property of the ALDH fold.

## Introduction

The aldehyde dehydrogenases (ALDH) catalyze the irreversible, NAD(P)^+^-dependent oxidation of aldehydes to their correspondent acids. They are dimeric or tetrameric proteins with a high degree of structural similarity but diverse physiological roles. Those with activity of betaine aldehyde dehydrogenase (betaine aldehyde: NAD(P)^+^ oxidoreductase, BADH) belong to different ALDH phylogenetic families and are distributed in bacteria, fungi, plants and animals, where they are involved in different physiological processes (reviewed in [1]). Some bacterial BADHs, as the ALDH9 from the opportunistic human pathogen *Pseudomonas aeruginosa* (PaBADH), participated in choline catabolism and may play an important role in the establishment and maintenance of the infection. Because of this, PaBADH has been proposed as a potential drug target [2], [3]. Others, as the ALDH10 from *Spinacia oleracea* (SoBADH), are involved in synthesis of the osmoprotectant glycine betaine in response to osmotic stress [4], [5]. The study of each of these two enzymes is of medical or biotechnological interest, but also their comparative study may shed light in important functional and structural characteristics in which they differ but share with other ALDH enzymes.

PaBADH is a homotetramer [6], [7] that become inactive when incubated in buffers of low ionic strength lacking K^+^ ions [6], [8], whereas SoBADH is a homodimer [5] that, retains its activity under the same conditions, as does another plant ALDH10 (the BADH from amaranth [6]). PaBADH and SoBADH are, therefore, representative of tetrameric and dimeric ALDHs, respectively, as well as of K^+^-dependent and K^+^-independent enzymes, respectively. Currently, only three other ALDHs are known to require K^+^ ions for activity: one ethanol-inducible, NAD^+^-dependent ALDH from *Pseudomonas spp*. [9] and two mitochondrial NAD(P)^+^-dependent ALDHs from *Saccharomyces cerevisiae* [10]–[12]. Other ALDHs are activated to some extent by K^+^ ions [6], [13]–[16] but as they do not require these cations for activity they should be considered K^+^-independent enzymes. The activation by K^+^ ions may be a more widespread feature of ALDHs, although only those enzymes known to be involved in the response to osmotic stress have been so far tested in this respect. The question of what makes the activity of an ALDH dependent on K^+^ ions has no answer yet.

**Figure 1:**
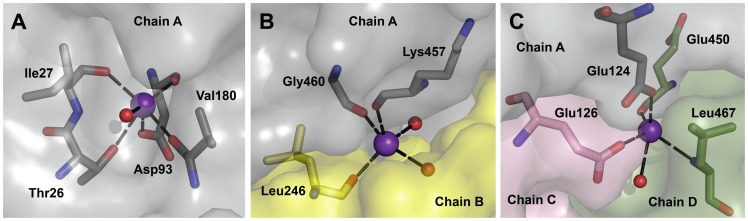
K^+^-binding sites of PaBADH. (**A**) The intra-subunit cation-binding site of subunit A. Similar sites exist in the other three subunits. (**B**) The inter-subunit cation-binding site observed between subunits A and B. Another symmetrical site exists between these two subunits, and there are two more between subunits C and D. (**C**) Central-cavity cation-binding site observed between subunits A, C and D. For clarity of the figure subunit B, which do not participate in this site, is not shown. Similar sites are present between subunits B, C and D, between subunits C, B and A, and between subunits D, B and A. K^+^ ions are shown as violet balls and water molecules as smaller red balls; amino acid side-chains are depicted as sticks with carbon atoms colored according to the subunit (grey for subunit A, yellow for B, pink for C, and green for D), oxygen in red and nitrogen in blue. Ion coordination bonds are depicted as black long-dashed lines. Images were generated using the PyMOL (http://www.pymol.org) and the PaBADH coordinates from the crystal of PDB accession code 2WME.

The effects of K^+^ ions on both the K^+^-dependent and the K^+^-independent ALDHs very likely result from different conformational states of the enzymes elicited by the specific binding of the cations. These structural effects were observed in K^+^-dependent enzymes as dissociation [17] and spectroscopic changes [18] in the absence of K^+^ ions, and as increased thermostability in their presence [19], [20]. Of the known four K^+^-dependent ALDHs, only the three-dimensional structure of PaBADH has been determined ([7], Protein Data Bank (PDB) accession codes 2WME, 2WOX, 2XDR, 3ZQA). A K^+^ ion was found in this crystal structure placed in a cavity at the surface of each subunit, making the same interactions with the protein than the Na^+^ ion found in the same region in human ALDH2 [21]. The PaBADH crystal structure also showed K^+^ ions bound at specific sites located in the subunit/subunit interfaces of the two dimeric units that form the tetramer [21], and in the central cavity formed by the four subunits where each cation is bridging three of the subunits [22] (Fig. 1). The intra-subunit and inter-subunit cation-binding sites were recently also found in the K^+^-independent SoBADH ([5], PDB accession code 4A0M), but this dimeric enzyme lacks the central-cavity site. A survey of the available crystal structures of ALDHs, as well as multiple sequence alignments of the known ALDH sequences, indicated that the intra-subunit sites are present in most of these enzymes, the inter-subunit sites in several of them, whereas the central-cavity sites are restricted to a few tetrameric enzymes [22]. Any or several of these cation-binding sites could be responsible for the observed effects of K^+^ ions on ALDHs.

In this work we aimed to further our understanding of the structural effects of K^+^ ions on ALDHs by carrying out a comprehensive analysis of the structural properties and thermal unfolding of PaBADH and SoBADH in the absence and presence of cations, using size exclusion chromatography (SEC), dynamic light scattering (DLS), far- and near-UV circular dichroism (CD) as well as intrinsic and extrinsic fluorescence. Although there are multiple studies on structural, kinetics and mechanistic aspects of many members of the ALDH superfamily, there are few, to the best of our knowledge, on the thermal stability of these enzymes [19], [20], [23]–[26] and on how ionic strength or cations affect this stability [19], [20]. Because of the high structural similarity between ALDHs, we anticipate that the results obtained in our comparative study of two enzymes that belong to different ALDH families and differ not only in amino acid composition but also in association state and in their dependence on K^+^ ions will be of relevance for the understanding of other members of this important group of enzymes.

## Materials and Methods

### Materials

1-Anilino-8-naphthalenesulfonic acid (ANS), dithiothreitol (DTT), thioflavin T (ThT), EDTA, and PIPES were obtained from Sigma-Aldrich. All other chemicals used in this study were of analytical grade.

### Expression, Purification and Assay of Recombinant PaBADH and SoBADH

PaBADH and SoBADH were expressed in *E. coli* cells as reported previously [2], [5] and purified to homogeneity following the procedures described [5], [8], [25]. The molar concentrations of the enzymes were determined spectrophotometrically, using molar absorptivity values at 280 nm deduced from their amino acid sequence by the method of Gill and von Hippel [27] (52,060 M^−1^ cm^−1^ for PaBADH and 43,200 M^−1^ cm^−1^ for SoBADH).

### Size Exclusion Chromatography

The association state of the enzymes under the several ionic conditions tested was estimated by SEC on a Superdex 200 HR 10/30 column connected to a high-pressure chromatography system (Waters, Milford, MA). The column was equilibrated and eluted at room temperature with three different buffers: 1 mM PIPES-tetraethylammonium (TEA) hydroxide, pH 6.9, containing 1 mM EDTA and 0.5 mM DTT (non-salt buffer) or the same plus 250 mM KCl (K-buffer) or 250 mM TEACl (TEA-buffer). Elution was performed at a flow rate of 0.5 ml/min and the eluted protein monitored by following absorbance at 280 nm. For calibration of the column, the following molecular size standards were employed: bovine thyroglobulin (670 kDa), bovine liver catalase (232 kDa), bovine gamma-globulin (158 kDa), horse myoglobin (17 kDa), and vitamin B12 (1.357 kDa). Before chromatography all samples were filtered (membrane-pore diameter 0.22 µm). SEC was also used to prepare samples in the different buffers immediately prior to the CD or fluorescence experiments. Due to a seven-fold dilution during the chromatographic run, the enzyme samples in the storage buffer were applied to the SEC column at appropriate concentrations to yield a concentration of the eluted protein higher than 0.25 mg/ml. The protein concentration was then adjusted to 0.25 mg/ml, which was the one used in the subsequent experiments unless otherwise indicated.

### Dynamic Light Scattering

Each protein sample was filtered (membrane-pore diameter 0.22 µm) shortly before acquiring its size distribution to remove any preexisting aggregates and dust particles. A 500 µl black quartz cuvette with 10 mm light path was used. Data were obtained with a Zetasizer µV DLS instrument equipped with a photodiode laser (830 nm) (Malvern Instruments Ltd., Worcestershire, UK). Dispersant refractive index and viscosity were assumed to be 1.33 and 1.003 cP, respectively. The temperature was maintained at 20°C during the measurements by means of a Peltier thermostating system. DLS values are averages of 10 distinct scans for 10 acquisitions over 10 s on a given sample. Instrument software was used to analyze the light scattering data.

### CD Spectroscopy

CD signals and dynode voltage were recorded with a Jasco J-715 spectropolarimeter (Jasco Inc., Easton, MD) equipped with a Peltier-type temperature control system (Model PTC-423S) and calibrated with *d*-10-(+)-camphorsulfonic acid. Near-UV (250–320 nm) and far-UV CD spectra (200–250 nm) were recorded for solutions of 0.25 mg/ml protein concentration placed in quartz cuvettes of 1.0-cm and 0.1-cm path length, respectively. Data were collected at 0.5 nm (near-UV) or 1.0 nm (far-UV) intervals, a bandwidth of 1.0 nm and at a scan rate of 20 nm/min. Spectra were averaged over 3–5 scans and the average spectrum of a reference sample without protein was subtracted. The observed ellipticities were converted to mean residue ellipticities [Θ] on the basis of a mean molecular mass per residue of 108.8 and 109.1 for PaBADH and SoBADH respectively. Near-UV CD spectra were also recorded as a function of ionic strength by incrementally adding aliquots of KCl or TEACl to a cuvette containing 0.25 mg/ml of PaBADH in the non-salt buffer at 20°C. Thermal-induced protein denaturation was monitored by following the changes in ellipticity at 222 nm by increasing the temperature from 20 to 90°C at a constant rate of 1.5°C/min. Determination of apparent *T*_m_ values was performed by non-linear regression fit of the data to a single or double Bolztman sigmoidal functions, as appropriated, and by differentiation of the curves using the Standard Analysis software provided with the instrument. ORIGIN software (OriginLab Corp.) was used for data analysis and display. The analysis of secondary structure content was performed with the deconvolution software CDPro [28], using SMP56 as the reference protein data set.

### Fluorescence Studies

The fluorescence spectra of enzyme solutions were measured in a spectrofluorometer ISS PC1 (Champaign, IL) equipped with a Peltier and water-jacketed cell holder for temperature control. Intrinsic tryptophan fluorescence spectra of PaBADH (0.25 mg/ml) were recorded with excitation wavelength set at 280 nm and bandwidths of 4 nm for both excitation and emission wavelengths. Extrinsic fluorescence spectra due to binding of 100 µM ANS to 0.25 mg/ml of enzymes were recorded from 400 to 560 nm with the excitation wavelength set at 360 nm. ThT binding was measured following the emission fluorescence of 25 µM ThT solution in the presence of 0.25 mg/ml PaBADH or SoBADH in the non-salt buffer at 20°C, before and after heating at 90°C. The fluorescence spectra were measured at an excitation wavelength of 448 nm and emission was recorded from 465 to 550 nm.

## Results

### Effects of Potassium and Ionic Strength on PaBADH and SoBADH Native Structure

**Figure 2:**
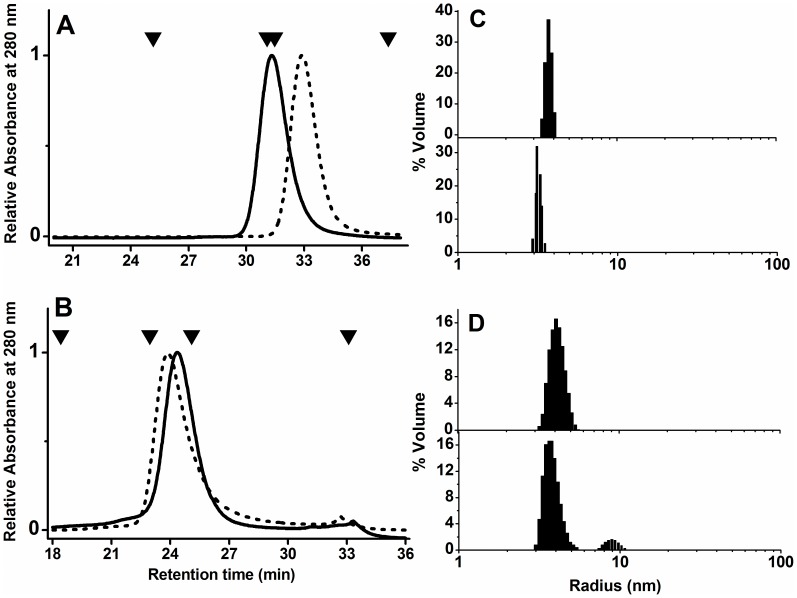
Effects of buffer ionic composition on the native structure of PaBADH and SoBADH. (**A** and **B**) SEC elution profiles of PaBADH (solid line) and SoBADH (dotted line) in 1 mM Pipes-TEAOH, pH 6.9, containing 1 mM EDTA and 0.5 mM DTT (non-salt buffer) (B), or in this buffer plus 250 mM KCl (K-buffer) (**A**). Arrows denote the positions of molecular-mass standards, from *left* to *right*: bovine tyroglobulin (670 kDa), bovine liver catalase (240 kDa), bovine gamma-globulin (158 kDa) and horse myoglobin (17 kDa). (**C** and **D**) DLS measurements. Hydrodynamic radius distribution plots of PaBADH (*top* of panels C and D) and SoBADH (*bottom* of panels C and D) in K-buffer (**C**) and non-salt buffer (D). Histograms are representative of volume-weighted size distributions of 0.25 mg/ml enzyme samples.

To distinguish the specific effects of potassium cations from non-specific ionic strength effects, the comparative studies carried out in this work were performed at low ionic strength in the absence of monovalent cations and in the presence of K^+^ or TEA^+^ ions, which are too bulky to specifically bind to the proteins. Therefore, cations effects on quaternary structure of PaBADH and SoBADH were studied by SEC and DLS at 20°C using three different buffers: a low-ionic strength buffer to which salt were not added (non-salt buffer); or this buffer plus 250 mM KCl (K-buffer) or plus 250 mM TEACl (TEA-buffer). In the K-buffer, at a protein concentration of 0.25 mg/ml, the concentration used in the CD and fluorescence experiments reported here, PaBADH and SoBADH eluted from the SEC column with retention times of approx. 30.5 and 32.7 min, which correspond to those of the tetrameric and dimeric enzymes, respectively (Figure 2A). Similar retention times were obtained when the proteins in the TEA-buffer were loaded on the column, but for both enzymes a small amount of soluble aggregates that eluted in the void volume were observed (data not shown). In the non-salt-buffer, under otherwise identical conditions, the elution profile of PaBADH showed a peak, with a retention time (24.1 min) much lower than that observed in the K-buffer, not because of protein aggregation but because of a decrease in electrostatic shielding by the buffer [29], which was also indicated by the calibration of the column with the molecular size standards (Figure 1C). In this condition, the retention time of SoBADH (23.6 min) indicates a significantly increase in its hydrodynamic radius, corresponding to the association of the enzyme into tetramers (Figure 1C).

DLS measurements were used to confirm the association state of these enzymes under the same conditions than those of the SEC experiments. The results, shown in Figure 2 and Table 1, indicate that PaBADH is mostly tetrameric regardless of the absence or presence of salt. The hydrodynamic radius (R_H_) values of PaBADH in the high-ionic-strength buffers (3.7 nm) were close to the theoretical gyration radius (R_G_) value of 3.75 nm calculated using the crystallographic coordinates of the tetramer and the program HYDROPRO [30]. In the non-salt buffer a R_H_ of 3.9 nm was obtained, slightly larger than the values determined in K- and TEA-buffers (Table 1). This suggests a more expanded conformation of the PaBADH tetramer in the low-ionic-strength buffer. The size distribution analysis of the DLS data of SoBADH in the non-salt buffer (Figure 2D and Table 1) gave two peaks. The major (94.5% of mass) had a mean R_H_ value of 3.6 nm, which is larger than the R_H_ value measured in K-buffer, and than the R_G_ value calculated for the dimer, but similar to the R_H_ value of native PaBADH tetramers. The minor (5.5% of mass) had a mean R_H_ value of 8.9 nm, which corresponds to soluble high-molecular mass aggregates. The DLS data of this enzyme in the TEA-buffer showed the formation of even larger aggregates with R_H_ ≥300 nm (data not shown), whereas in the K-buffer the experimental R_H_ value, 3.1 nm, is the same as the theoretical R_G_, indicating the dimeric nature of SoBADH in the presence of K^+^ ions. In conclusion, both SEC and DLS data clearly indicate that K^+^ ions favour the native structure of both proteins and that PaBADH is an inactive expanded tetramer in the absence of cations, while the dimeric SoBADH associates forming active tetramers.

**Table 1:** DLS data of PaBADH and SoBADH under different salt conditions.

Enzyme	Condition	Mass (%)	Polydispersity (%)	R_H_ a (nm)	R_G_ b (nm)
PaBADH					3.75
	Non-salt buffer	99.9	14±6	3.9±0.1	
	K-buffer	100	9±4	3.7±0.1	
	TEA-buffer	99.9	8±0	3.7±0.1	
SoBADH					3.09
	Non-salt buffer	94.5	21±6	3.6±0.2	
	K-buffer	99.9	7±3	3.1±0.1	

a The mean radius corresponds to the highest peak of the size distribution histograms shown in Figure 1.

b Calculated using the program HYDROPRO (version 10) and the atomic coordinates of PaBADH (PDB accession code 2WME) or SoBADH (PDB accession code 4A0M).

**Figure 3:**
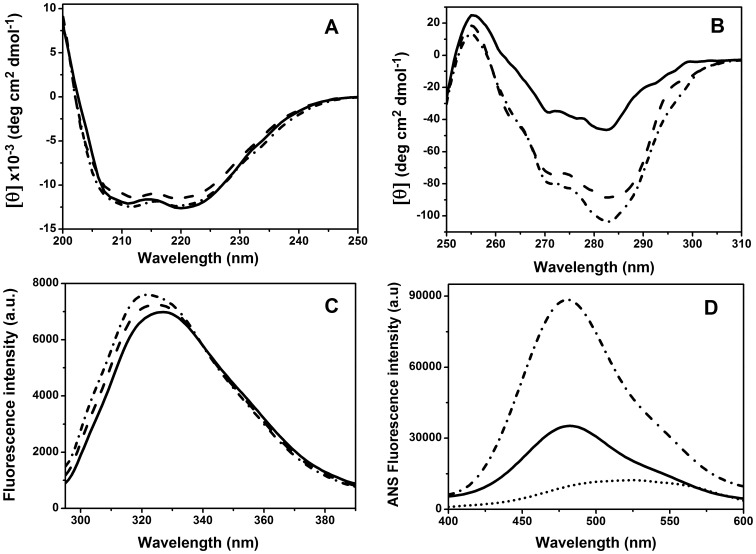
Effects of buffer ionic composition on the conformational characteristics of PaBADH. (**A** and **B**) Far- and near-UV CD spectra. (**C** and **D**) Intrinsic and ANS fluorescence emission spectra. Enzyme samples (0.25 mg/ml) were in non-salt buffer (solid lines), K-buffer (dotted and dashed lines), or TEA-buffer (dashed lines). In (**D**) the spectrum of ANS in the absence of enzyme is shown as a dotted line. Spectra were recorded at 20°C.

The far-UV CD spectra of PaBADH in the three different buffers were almost indistinguishable, indicating that the secondary structure of the protein was nearly identical regardless of the presence or absence of monovalent cations in the solution (Figure 3A). The spectra showed two clear negative bands around 208 and 222 nm in agreement with the α-helical content of this enzyme showed by its crystal structure [7]. The near-UV CD spectrum of PaBADH in K-buffer was dominated by a mayor negative band at 282 nm, corresponding to Tyr residues. A shoulder was observed at 270 nm, which is attributed to Phe residues. Although PaBADH has six Trp residues per subunit, a defined band in the 285–300 nm region was not observed; only a small shoulder around 286 nm can be associated with the Trp signal (Figure 3B). The second derivative of a near-UV CD spectrum obtained at 1 mg/ml was used for resolving overlapping peaks caused by the large Tyr signal at 282 nm. The hidden bands at 288 and 293 nm that can be ascribed to Trp signals were observed in this way (Figure S1). Contrary to the lack of effects of the absence of the cation on secondary structure, the tetrameric PaBADH in the non-salt buffer exhibited a significant decrease (more than 50%) in the magnitude of the near-UV CD signal (Figure 3B) and a weakening of the Trp bands at 288 and 293 nm (Figure S1B). In the TEA-buffer the ellipticity values around 270–295 nm were higher than in the non-salt buffer but still lower than those obtained in the K-buffer (Figure 3B), suggesting that K^+^ ions have specific structuring effects on the PaBADH tetramer, additional to those of ionic strength. This conclusion is also supported by the changes in the asymmetry of the environment of some tryptophan residues observed in the absence of salt and particularly of K^+^ cations (Figure S1B).

The observed differences in the near-UV CD spectra correlate with those observed by following the intrinsic fluorescence emission spectrum of the PaBADH protein, which in the non-salt buffer exhibited a clear red shift (of 5 nm at the wavelength of maximal emission, and about 2.8 nm of the SCM) as well as a moderate decrease in fluorescence intensity relative to that of the protein in the K-buffer (Figure 3C). A similar, though smaller, change was seen in the fluorescence spectrum of the protein in the TEA-buffer, also indicating loses in PaBADH structure caused by the absence of K^+^ ions. These results confirm that the PaBADH tetramer that exists under low ionic strength conditions has less tertiary structure than the tetramer existing at higher ionic strength, although it retains most of the secondary structure.

To further explore the effects of K^+^ ions on PaBADH tertiary structure, we determined the ANS binding to this enzyme in the non-salt and K-buffers under the same conditions than above, i.e., 20°C and 0.25 mg protein/ml. ANS has been extensively used in monitoring hydrophobic patches or cavities on the surface of proteins exposed as a consequence of either unfolding or of conformational changes [31]. When binding to hydrophobic surfaces, the fluorescence intensity of the probe increases and shifts its emission maximum to a lower wavelength depending on the apolar nature of its microenvironment. In the non-salt buffer (Figure 2D) there was an enhancement of the ANS fluorescence emission intensity in the presence of the protein (3.6-fold) as well as a blue shift in the emission maximum wavelength (from 522 nm in the free ANS to 488 nm in the ANS plus protein sample). PaBADH in the K-buffer produced an even greater increase in ANS fluorescence intensity (10-fold) and blue shift (to 480 nm), indicating greater binding of ANS in the presence of K^+^ ions than in their absence. Since in the absence of protein the fluorescence of ANS is not affected by 250 mM KCl, the latter result suggests that there are more structured protein regions with affinity for ANS in the native tetramer than in the non-native one present at low ionic strength, Alternatively, the enhanced binding may be caused by non-specific effects of ionic strength favoring the hydrophobic interactions between ANS and the protein. The latter possibility could not be tested with TEACl because TEA^+^ ions increased the fluorescence of the probe in the absence of protein. We do not have yet any structural evidence of the nature of the possible ANS-binding regions of the native tetrameric PaBADH.

In regard to SoBADH, its secondary and tertiary structures were not significantly affected by the lack of cations in the incubation buffer and showed minor changes in the non-salt buffer relative to the K-buffer, notwithstanding that it associates into a tetrameric form in the non-salt-buffer, as shown by SEC and DLS experiments. Under both salt conditions, the far-UV CD and ANS fluorescence spectra were similar (Figure S2A and S2C). Different from PaBADH, the near-UV-CD spectrum of SoBADH showed well-defined positive peaks at 283 and 291 nm and a low, but reproducible, increase (20%) in the signal intensity in the non-salt buffer compared to the spectrum in the K-buffer (Figure S2B). The latter finding indicates that at low ionic strength and absence of potassium the conformation of the SoBADH protein was much less affected than that of PaBADH.

**Figure 4:**
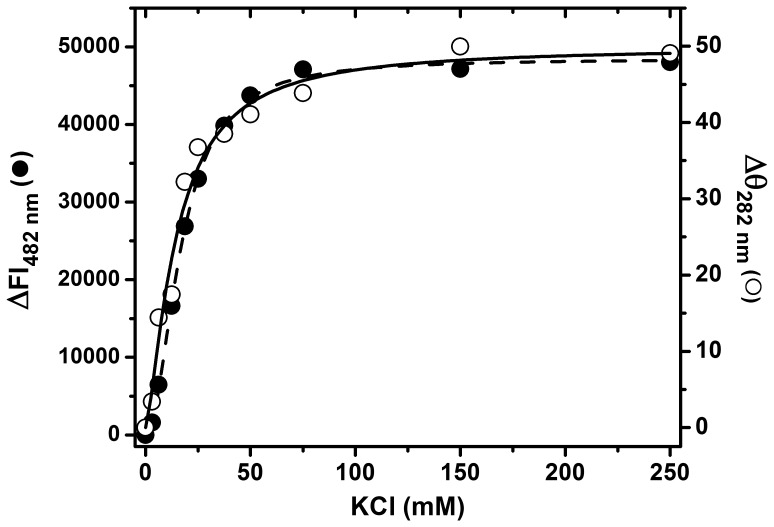
PaBADH conformational changes induced by the progressive increase in KCl concentration in the incubation medium. Enzyme samples (0.25 mg/ml) in non-salt buffer were incubated at 20°C with increasing concentrations of KCl; after 3 min equilibration, the near-UV CD spectra and the fluorescence emission spectra of ANS were recorded. Changes in the ellipticity value at 280 nm of the near UV-CD spectra (open circles) and in fluorescence intensity at 482 nm in the emission spectra of ANS (closed circles) are plotted as a function of KCl concentration. The points represent the absolute value of the observed changes and the curves correspond to the fit of the data to the Hill equation by nonlinear regression.

Increasing KCl concentrations, in a range from 3.125 to 150 mM, induced progressive increases in PaBADH mean residue ellipticity between 260 and 290 nm (Figure S3A) as well as in the fluorescence intensity of bound ANS (Figure S3B), until the values characteristic of the native tetramer are reached. The dependency of the extent of PaBADH conformational changes, followed either by near-UV CD signal or ANS fluorescence, on KCl concentration showed saturation by the cation. Plots of ellipticity and ANS fluorescence emission intensity data *versus* KCl concentration (Figure 4) were best fitted to a Hill equation, giving values for the cation concentration that produces half of the maximum change of 14.6 (±1.6) and 16.9 (±0.3) mM, respectively. These values are similar to those of the dissociation constant (*K*_d_) of protein-cation complexes reported for the specific binding of monovalent cations to other proteins [32]–[34] but higher that those estimated for the yeast K^+^-dependent ALDHs, which were reported to be around 2–6 mM [18], [34]. Binding of K^+^ ions to PaBADH was cooperative, as indicated by Hill numbers of 1.41 (±0.22) and 1.99 (±0.07) estimated for the far-UV CD and ANS data, respectively. These results support that the specific binding of K^+^ ions to one cation-binding site is responsible for the changes in tertiary structure of PaBADH, and indicate that these structural changes are reversible, in agreement with the previously observed reversibility of the loss of PaBADH activity elicited by the absence of potassium [6].

### Effects of Potassium and Ionic Strength on the Thermal Denaturation of PaBADH and SoBADH

In a first approach, the thermal-induced unfolding transitions of the enzymes were monitored by following the far-UV CD signal at 222 nm, which indicates the α-helical content of the protein. Under all conditions tested, thermal denaturation of both enzymes was irreversible, thus precluding equilibrium thermodynamic analysis. However, the use of the same measurement parameters and of the same experimental conditions, other than the nature or concentration of cations, enabled us to compare the effect of cations and ionic strength on the enzymes stability. Moreover, despite this irreversibility, thermal transitions curves and apparent *T*_m_ values were reproducible.

**Figure 5:**
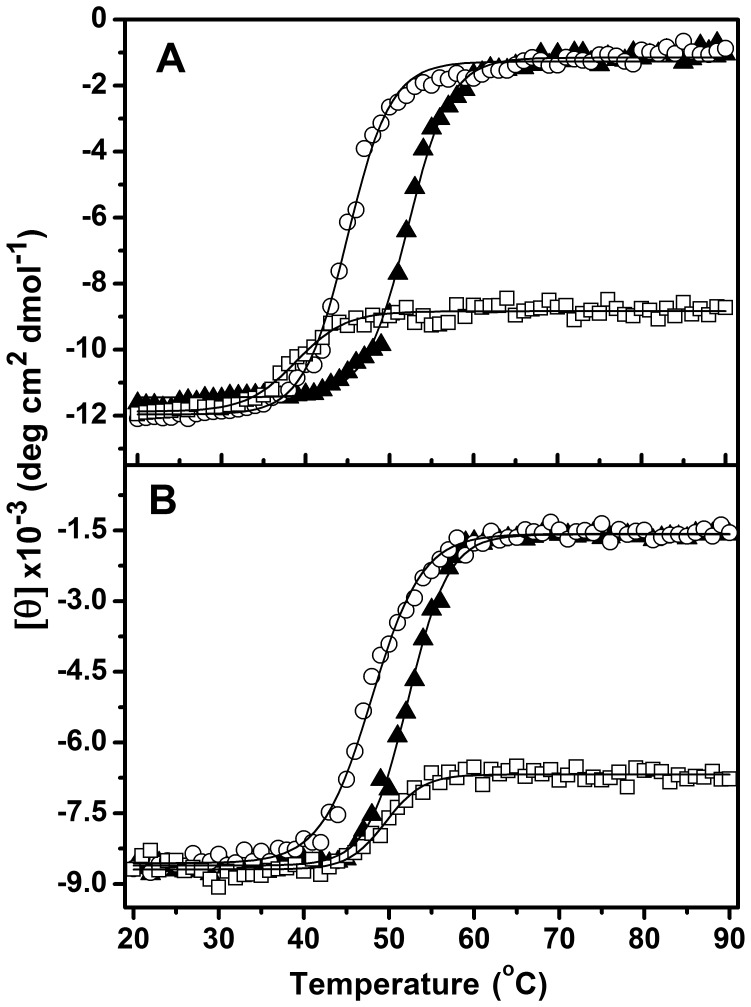
Effect of salts on the thermal denaturation of PaBADH and SoBADH. Heat-induced transitions of PaBADH (**A**) and SoBADH (**B**) samples (0.25 mg/ml) in the non-salt buffer (squares), K-buffer (open triangles) or TEA-buffer (open circles) were determined by following the changes at 222 nm in the far-UV CD signal. The temperature range was 20–90°C and the scan rate 1.5°C/min. Transitions were evaluated with a Boltzmann function.

A complete loss of the ellipticity signal and highly cooperative thermal transitions were observed in K- and TEA-buffers (Figure 5), but the estimated apparent *T*_m_ values in the presence of 250 mM KCl, were consistently higher than in the presence of 250 mM TEACl (51.8 *versus* 44.9°C for PaBADH and 51.2 *versus* 47.9°C for SoBADH). These findings indicate that K^+^ ions have specific stabilizing effects on both enzymes, although the effect is greater on PaBADH. After heating in both buffers, the denatured enzymes formed visible, non-soluble aggregates that precipitated. To measure the thermal-induced aggregation of the proteins, sample turbidity was followed in the CD experiments by recording changes in dynode voltage (Figure S4). As the temperature was raised, the initial decreases in the far-UV CD signal were not accompanied by significant changes in voltage, indicating losses of secondary structure without protein aggregation. But at higher temperatures there were clear increases in turbidity that closely correlated with the thermal transitions monitored by far-UV CD, indicating that aggregation occurs simultaneously to unfolding at these temperatures. The following decreases in turbidity observed at even higher temperatures are due to precipitation of large protein aggregates.

In the non-salt buffer, a sigmoidal thermal transition was also obtained with both PaBADH and SoBADH but, interestingly, only 20–25% of the CD ellipticity at 222 nm was lost after heating up to 90°C (Figure 5). This partial loss of ellipticity was not caused by protein precipitation, as indicated by relatively small dynode voltage increases (Figure S4). Under this condition, PaBADH had an apparent *T*_m_ value of 40±0.7°C, which was 5 and 12°C lower than the apparent *T*_m_ values determined in the presence of TEA^+^ or K^+^ ions, respectively, indicating the important stabilizing effects that K^+^ ions have on this enzyme and also a significant stabilizing effect of either the ionic strength or of TEACl. In contrast, the estimated apparent *T*_m_ of the thermal transition of SoBADH in the non-salt buffer, 49.6±2.2°C, was only 2.0°C lower than in the K-buffer. It is to be noted that in the presence of a near physiological concentrations of K^+^ ions the tetrameric PaBADH and the dimeric SoBADH exhibited a similar thermostability, which suggests that the stability of both enzymes has been optimized, regardless of their association state.

**Figure 6:**
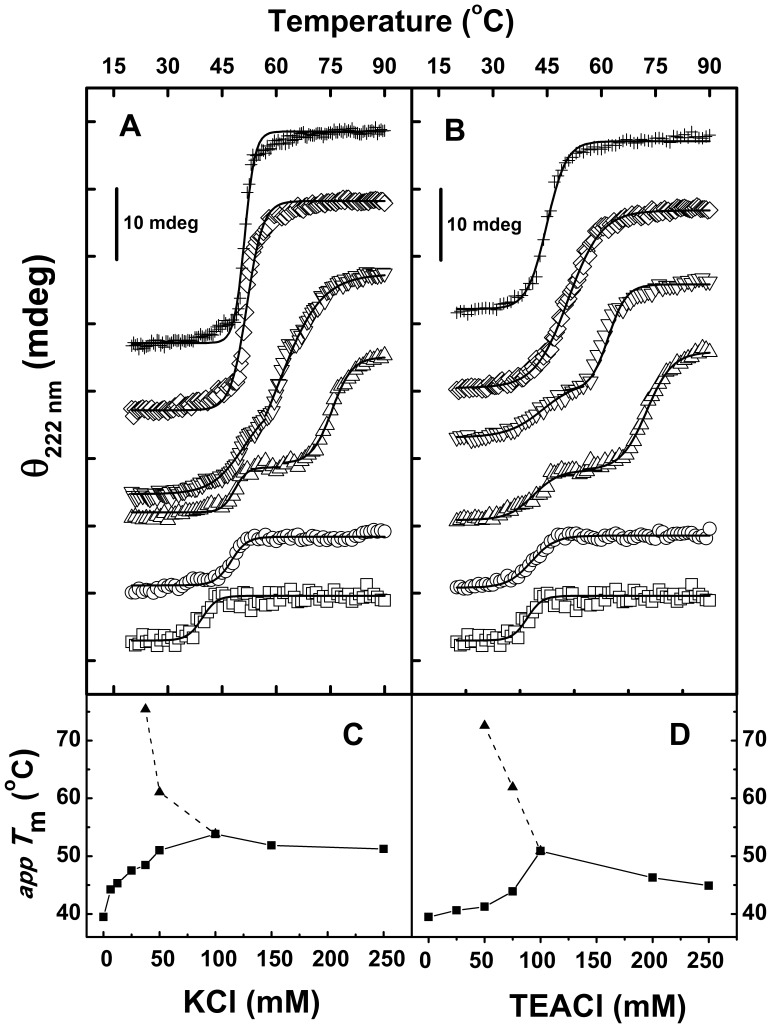
Effects of the concentration of monovalent cations on the thermal denaturation profiles of PaBADH. Temperature-induced changes in CD ellipticity at 222 nm were measured at increasing concentrations of cations. The thermal profiles shown have been shifted 10 mdeg over the *y-*axis for clarity of the figure. From bottom to top: Enzyme samples (0.25 mg/ml) incubated in the non-salt buffer (open squares) or in the presence of 25, 37.5, 50, 150 and 250 mM KCl **(A),** or of 25, 50, 75, 100 and 250 mM TEACl (**B**). The thermal profile at zero salt starts at –27.01 mdeg and ends at –20.37 mdeg. Changes in the temperature of thermal transition (solid squares) at increasing KCl **(C)** or TEACl concentrations (**D**). In (**C**) and (**D**) the *T*_m_ values of the second thermal transitions (solid triangles) observed at 37.5 and 50 mM KCl or at 50 and 75 mM TEACl are also shown. The temperature range was 20–90°C and the scan rate was 1.5°C/min. The solid lines in **(A)** and **(B)** represent the best fit of the single or double thermal transition data to a single or double sigmoidal Boltztmann functions, respectively, by non-linear regression analysis.

To understand the mechanism underlying the K^+^ ions effects on the thermal stability of both enzymes, we carried out thermal denaturation studies using buffers with increasing concentrations of KCl. At 6.5, 12.5 and 25 mM KCl concentrations, the thermal unfolding profiles of PaBADH were similar to the one observed in the non-salt buffer, i.e., there was a loss of only 20–25% CD ellipticity at 222 nm associated with the thermal denaturation (Figure 5A) and no protein precipitation was observed after heating. But the apparent *T*_m_ values were 4 to 7.5°C higher than the one determined in the absence of K^+^ ions (Figure 6C). At 37.5 and 50 mM KCl two distinct thermal transitions were observed: the first leading to a partial loss of secondary structure, similar to the transitions observed at lower salt concentrations; the second leading to the complete loss of the CD signal that corresponded with precipitation of the protein (Figure 6A). The increases in salt concentration had opposite effects on both transitions so that eventually the low and high thermal transitions converged on the same apparent *T*_m_ value of approximately 51°C. A plot of the apparent *T*_m_ values *versus* KCl concentration shows a hyperbolic dependence of the PaBADH thermal stability on K^+^ ions concentration (Figure 6C), supporting that the effects of the cation were achieved through its specific binding to the protein. Moreover, the K^+^ ion concentration that gives half of the maximum change on the apparent *T*_m_ was similar to the concentration estimated using the CD and ANS binding data shown in Figure 3.

Increasing concentrations of TEA^+^ ions also increased the apparent *T*_m_ of the first thermal transition of PaBADH but in a significantly lower extent than K^+^ ions did. Indeed, at low TEACl concentrations the apparent *T*_m_ values of the first transition remained almost unchanged with respect to the value in the non-salt buffer (Figure 5B and 5D). As observed with KCl, at intermediate concentrations of TEACl there were two distinct thermal transitions, which eventually converged on a single transition when the salt concentration was increased further.

The thermal unfolding profiles of SoBADH followed by far-UV CD changed in the same manner as those of PaBADH when the K^+^ ions concentration were increased (Figure S5). At 12.5 mM KCl the thermal unfolding profile and the apparent *T*_m_ were similar to those observed in the non-salt buffer, two transitions were observed at 25 mM KCl, and a single transition with an apparent *T*_m_ value about 51.0°C was observed at 37.5 and 250 mM KCl. These results confirmed that K^+^ ions also have stabilizing effects against thermal denaturation of SoBADH, as indicated by the increases in the apparent *T*_m_ values as the K^+^ ions concentration increases (inset Figure S5), although they stabilize this enzyme in a lesser degree than PaBADH as mentioned above.

Together, the results with both enzymes indicate that the non-native, structured protein species formed by heating at low salt concentrations are destabilized by an increase in ionic strength, which favour their aggregation by high temperatures, as indicated by dynode voltage changes (not shown). When the KCl or TEACl concentrations were raised, the extent of aggregation of both proteins was greater and protein precipitation occurred at increasingly lower temperatures.

### Formation of a Molten-globule-like Intermediate during Heat Denaturation of PaBADH

**Figure 7:**
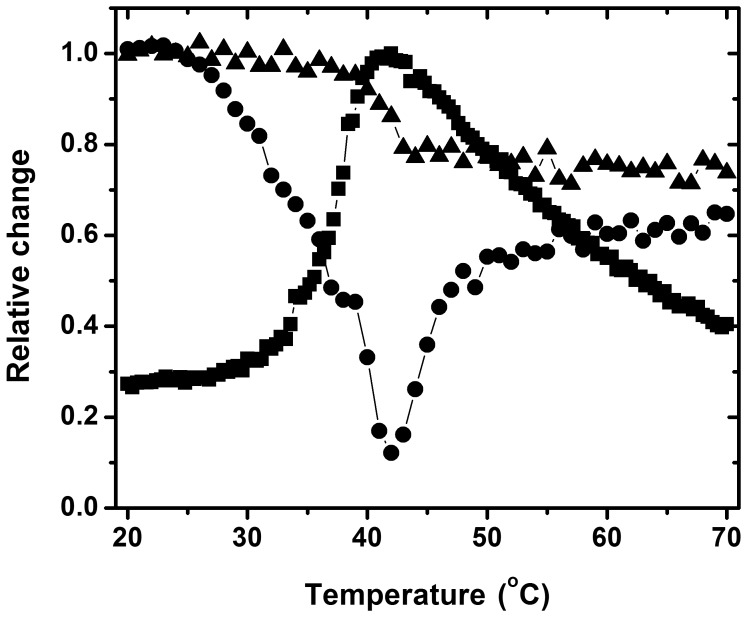
Secondary and tertiary structural changes induced by temperature at low ionic strength on PaBADH. The thermal denaturation of the enzyme (0.25 mg/ml) in the non-salt buffer was monitored by changes in ellipticity in the far-UV CD at 222 nm (closed triangles), near-UV CD at 282 nm (closed circles), and ANS fluorescence emission intensity at 482 nm (closed squares). The temperature range was 20–90°C and the scan rate 1.5°C/min. The CD signals are represented as fraction of the values observed at 20°C. The ANS signal was normalized using the maximum fluorescence intensity observed at 42°C.

When the thermal denaturation of PaBADH was followed by changes of ANS fluorescence, a maximum of ANS binding was observed at any of the K^+^ ions concentrations tested (Figure S6). These results indicate that a partially unfolded intermediate that exposes hydrophobic surfaces forms at the beginning of every thermal transition. The temperature at which the ANS fluorescence maximum occurs increased as the K^+^ ions concentration increased, again supporting the stabilizing effects of these cations on PaBADH. To gain insight into the conformational properties of this unfolded intermediate we followed the thermal denaturation of PaBADH in the non-salt buffer by near-UV CD and ANS fluorescence (Figure 6). The thermal transition followed by the CD signal at 222 nm took place in a temperature range of 37 to 44°C, with an apparent *T_m_* of 41°C, but the beginning of the thermal disruption of the tertiary structure preceded the thermal loss of secondary structure by approximately 10°C. Temperature increases from 27 to 42°C were accompanied by a gradual decrease in the CD signal at 282 nm, indicating a collapse of the tertiary structure that correlates with the partial loss of secondary structure. At temperatures higher than 42°C, a gradual increase in the 282 nm ellipticity signal was observed, resulting in a significant recovery of the near-UV CD signal, which reached approximately 60% of that of the native enzyme, without changes in the far-UV CD signal (Figure 7). Measurements of the temperature dependence of ANS fluorescence intensity at 482 nm showed that the intensity maximum occurs at the temperature at which the corresponding near-UV CD spectrum showed the end of the thermal transition that result in the collapse of the tertiary structure. At temperatures higher than 42°C a gradual decrease in ANS binding was observed. Thus, under the non-salt conditions, the changes in the near-UV CD and ANS fluorescence signals showed two temperature-induced conformational transitions of PaBADH: the first leading to a molten globule-like intermediate, characterized by retaining considerable secondary structure, few tertiary structure, increased ANS binding and proneness to aggregate; the second leading to a soluble, non-native structured protein, which is maintained even at 90°C in the absence of salt but that aggregates in its presence.

### Conformational Properties of the Structured Non-native Protein Species Formed by Heating at Low Ionic Strength

**Figure 8:**
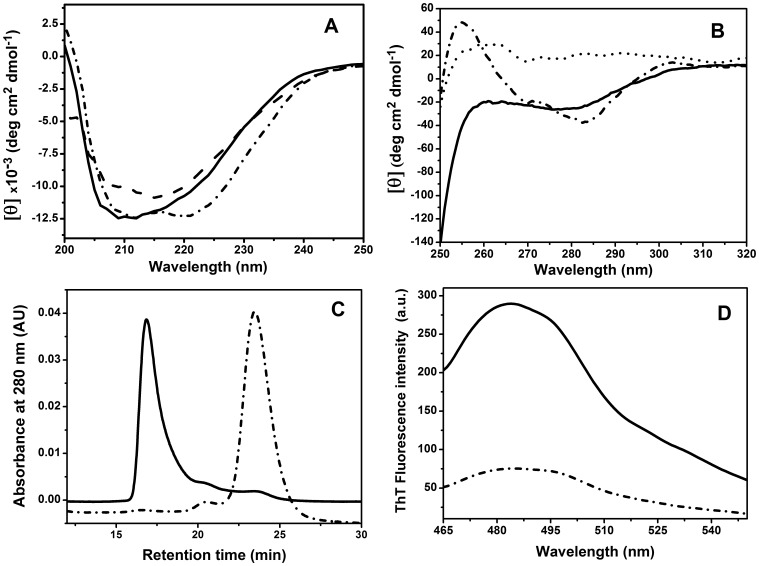
Structural properties of PaBADH thermally denatured in the non-salt buffer. (**A**) Far-UV CD spectra of heat-denatured protein at 90°C (long-dash lines) and after cooling back to 20°C (solid lines). (**B**) Near-UV CD spectra of the enzyme after cooling back to 20°C (solid lines), and after denaturation with 6 M guanidinium chloride (dotted line). Enzyme samples (0.25 mg/ml) were heated at a rate of 1.5 min/°C. (**C**) SEC elution profile of enzyme (0.25 mg/ml) after being heated at 37°C for 2 h (solid line). (**D**) ThT fluorescence emission spectrum of the sample heated at 90°C and cooled back to 20°C (solid line). With comparative purposes, the CD and ThT fluorescence spectra as well as the SEC elution profile of the same samples obtained at 20°C before heating are shown as dash-dotted lines.

As mentioned above, thermal denaturation of PaBADH and SoBADH followed by far-UV CD in non-salt or low-salt buffers showed a single transition that resulted in approximately 20% loss in secondary structure. To gain insight into the conformational properties of the non-native, structured protein species formed in this transition, we obtained their far-UV CD spectra after being heated up to 90°C and after being cooled down to 20°C. This could be done because both proteins remained soluble when heated in the non-salt buffer. The spectrum of PaBADH at 90°C (Figure 8A) confirmed the partial unfolding of the protein after thermal denaturation in this condition. Deconvolution of the far-UV CD spectra of the non-heated, heated, and heated-cooled PaBADH samples indicates that there was a loss of α-helical content (from 38% in the native enzyme to 20%) and a gain in β-structure (from 15 to 30%) after thermal denaturation (Table 2). Although the heated protein can partially recover its α-helical content after cooling to 20°C, the native secondary structure was not fully regained, resulting in higher β-sheet content. Similar results were obtained with SoBADH (Table 2). In addition, the protein tertiary structure of PaBADH was noticeable changed but not totally lost, as judged by comparing the near-UV CD spectrum of the protein that had been heated at 90°C and then cooled to 20°C with the spectra of the non-heated or guanidinium chloride denatured proteins (Figure 7B). The structured PaBADH species that result after heating and cooling in the absence of salt eluted in the void volume of the SEC column (Figure 7C), showing that they are soluble aggregates.

**Table 2:** Effect of heating at low ionic strength on the secondary structure^a^ of PaBADH and SoBADH^b^.

Temperature (°C)	20	90	20 (cooled from 90)
Secondary structure (%)			
α-Helix	38 (37)	20	32 (17)
β-Sheet	15 (19)	30	25 (29)
Turns	19 (17)	19	19 (23)
Unordered	28 (27)	31	24 (31)

a Estimated using the deconvolution software CDPro [28].

b SoBADH values are given in parenthesis.

In some proteins, heat-induced unfolding leads to formation of β-sheet fibrillar aggregates that resemble amyloids [35]–[37]. These aggregates have the ability to bind the fluorescent dye ThT, causing a major increase in the fluorescence emission intensity of the dye around 480 nm [38]. The ThT fluorescence signals at 478–484 nm of a PaBADH solution in the non-salt buffer after being heated at 90°C and cooled back to 20°C were significantly higher than those of the non-heated protein (Figure 8D), indicating the binding of the dye to the PaBADH soluble aggregates. This finding is in agreement with the increases in β structure observed in the far-UV CD spectrum. Heat treatment of SoBADH in the non-salt condition also led to formation of soluble aggregates that bind ThT (results not shown).

## Discussion

### Structural Bases for the Different Effect of K^+^ Ions on PaBADH and SoBADH Structure

PaBADH and SoBADH have two kinds of K^+^ ions binding sites: the intra-subunit and the inter-subunit ones [5],[7]. Binding of K^+^ ions to one or both of these sites would explain the observed stabilizing effects of these cations on SoBADH and part of their stabilizing effects on PaBADH. However, it seems that there is not a correlation between having these cation-binding sites and the enzyme activity being K^+^-dependent or K^+^-independent, as both PaBADH (K^+^-dependent) and SoBADH (K^+^-independent) have these sites. The quantitatively and qualitatively different effects of potassium on these enzymes might be due to the central-cavity K^+^ ion-binding sites, which do not exist in the dimeric SoBADH. In each of these sites the K^+^ ion bridges three of the PaBADH subunits, thus contributing to the stability of the tetrameric native structure of the enzyme, Moreover, in the absence of a cation bound in the latter sites the PaBADH tertiary structure will be greatly affected because of electrostatic repulsion between the two close carboxylic groups of the side-chains of Glu124 of one subunit and Glu126 of a neighbor subunit (Figure 1). This will lead to disruption of the actives sites located in the immediacy of the central cavity, thus accounting for enzyme inactivation. A major contribution of the central cavity K^+^-binding sites to the stability of PaBADH also explains the cooperative binding of the K^+^ ions observed in the present work, since their binding to one of these sites will favor the correct structuring of the others and therefore the binding of cations to empty central-cavity sites. Among the known ALDHs, a central-cavity K^+^-binding site with the same characteristics than the one observed in PaBADH only exists in ALDH9 enzymes from the *Pseudomonas* genera [22], suggesting that these enzymes will be K^+^-dependent for the same reasons that PaBADH. A variation of the central-cavity sites exists in fish and amphibian ALDH9s and in ALDH1Ls [22], but since no carboxyl group participates in forming these sites, it can predicted that the absence of K^+^ ions will not have the important structural and functional consequences observed in PaBADH. Interestingly, the crystal structures of some ALDH5 and ALDH11 enzymes have the central-cavity glutamyl residues in a similar arrangement than the one found in PaBADH crystallographic structures, but in these enzymes the negative charges of the carboxyl groups are neutralized by the side-chain amine group of a lysine that occupies the position of the cation in PaBADH [22]. This confirms the need of a positive charge in this position when two carboxyl groups are present side by side.

In PaBADH, the central cavity is connected to the protein surface by four winding tunnels. In fact, the inter-subunit K^+^-binding sites are in a depression of these tunnels, a few angstroms away from where they end in the central cavity. The size of the tunnels allows the passage of even bulky cations, as TEA^+^, through them, which probably explains our finding that TEA^+^ ions can also stabilize the PaBADH structure to some extent, But the effects of TEA^+^ ions are limited to the neutralization of negative charges in the central cavity since they cannot properly bind to any of the four K^+^ ion-binding sites. Probably, this is why TEA^+^ ions are not as effective as K^+^ ions in the thermal stabilization of the enzyme.

### Formation of Soluble, Structured Aggregates by Heating PaBADH and SoBADH at Low Ionic Strength

When heated at low ionic strength, PaBADH and SoBADH form similar soluble aggregates in spite of their differences in amino acid composition (phylogenetically these enzymes are no closer to each other than to any other member of the ALDH superfamily), electrostatic potential surface (not shown) and oligomerization state. These soluble aggregates are heat resistant, retain considerable secondary structure, have an increased β-sheet content and bind ThT. All these features are consistent with β-sheet pre-fibrillar aggregates. The enhancement of ThT fluorescence intensity is not restricted to complete amyloid fibrils but also occur in the presence of small soluble aggregates [36], [39], [40] as the ones formed in PaBADH and SoBADH by heating in a low-salt medium. It is known that many proteins, even from organisms that not are subjected to amyloid deposition diseases, are able to form amyloid oligomers, but to the best of our knowledge, this is the first report of β-sheet pre-fibrillar aggregates in the ALDH superfamily.

Since all ALDH enzymes of known three-dimensional structure, either dimeric or tetrameric, have high structural similarity and the same fold [41], this response to heat and ionic strength may indeed be a general property of the members of the ALDH superfamily, related with their particular fold. ALDH monomers consist of three domains: an N-terminal coenzyme binding domain, which contains a six-stranded β-sheet, five of the strands parallel and one antiparallel; a catalytic domain, containing a seven-stranded parallel β-sheet; and an oligomerization domain formed by a three-stranded antiparallel β-sheet, which interact with the other monomer on one side but is exposed to the surface on its other side. In addition there is a small two-stranded antiparallel β-sheet in the surface or each monomer (Figure S7A). The three β-strands of the oligomerization domain of one monomer together with the seven β-strands of the catalytic domain of the other monomer in a dimer form a ten-stranded β-sheet, so that in tetrameric ALDHs two 20-stranded β-sheets are formed [41]. ALDH enzymes may be prone to form amyloid-like aggregates as a consequence of their particular structural arrangement of these β-sheets. In the native structure of the ALDH enzymes, superficial α-helices or loops cover the β-strands of the catalytic domain and part of those of the nucleotide domain (Figure S7B), preventing their interaction with those of other molecules. It can be speculated that increases in temperature at low ionic strength disorganize any of these regions, exposing the β-strands and allowing formation of soluble β-aggregates.

## Supporting Information

Figure S1 **Effects of cations on the near-UV CD spectra of PaBADH.** (**A**) Enzyme samples (1 mg/ml) were in 1 mM Pipes-TEAOH, pH 6.9, containing EDTA 1 mM and 0.5 mM DTT (non-salt buffer; black line), in the non-salt buffer plus 250 mM KCl (K-buffer; red line), or in non-salt buffer plus 250 TEACl (TEA-buffer; blue line). Spectra were recorded at 20°C. (**B**) Second derivative of the spectra shown in (**A**). a.u., arbitrary units.
(TIF)

Figure S2 **Effects of K^+^ ions on the conformational characteristic of SoBADH.** (**A** and **B**) Far- and near-UV CD spectra. (**C**) ANS fluorescence emission spectra. Enzyme samples (0.25 mg/ml) were in non-salt buffer (black lines), K-buffer (red lines), or TEA-buffer (dashed lines). In (**C**) the spectrum of 100 µM ANS in the absence of enzyme is shown as a black dotted line. Spectra were recorded at 20°C.
(TIF)

Figure S3 **PaBADH conformational changes induced by the progressive increase in KCl concentration in the incubation medium.** Enzyme samples (0.25 mg/ml) in non-salt buffer were incubated at 20°C with the concentrations of KCl indicated in the figure; after 3 min equilibration, the near-UV CD spectra (**A**) and the fluorescence emission spectra of 100 µM ANS (**B**) were recorded. a.u., arbitrary units.
(TIF)

Figure S4 **Effects of salts on the aggregation of PaBADH and SoBADH during thermal denaturation.** PaBADH (**A**) and SoBADH (**B**) samples (0.25 mg/ml) in non-salt buffer (black lines), K-buffer (red lines) or TEA-buffer (blue lines) were incubated in the spectropolarimeter and the changes in dynode voltage were registered. The temperature range was from 20 to 90°C and the scan rate 1.5°C/min. The apparent *T*_m_, values determined in the same experiments by following the changes at 222 nm in near-UV CD spectra (shown in Figure 4 of the main article) are indicated by arrows. The increases in voltage observed in K- and TEA-buffers are due to protein aggregation that occurs simultaneously to the thermal transition. The decreases in voltage at temperatures above the apparent *T*_m_ values reflect precipitation of the heat-aggregated proteins.
(TIF)

Figure S5 **Effects of the concentration of K^+^ cations on the thermal denaturation profiles of SoBADH.** Temperature-induced changes in CD ellipticity at 222 nm were measured at increasing concentrations of K^+^ cations. The thermal profiles shown have been shifted 10 mdeg over the *y-*axis for clarity of the figure. From bottom to top: Enzyme samples (0.25 mg/ml) incubated in the non-salt buffer (open squares) or in this buffer plus of 12.5 (open circles), 25 (open triangles), 37.5 (open rhombuses), and 250 (crosses) mM KCl. The thermal profile at zero salt starts at –27.01 mdeg and ends at –20.37 mdeg. Inset: Dependency of the apparent *T*_m_ values on KCl concentration. The apparent *T*_m_ value of the second thermal transition observed at 25 mM KCl is shown as a solid triangle. The temperature range was 20–90°C and the scan rate 1.5°C/min. The solid lines represent the fits of the data to a single or double Boltztman equation, as appropriate, by non-linear regression analysis.
(TIF)

Figure S6 **Effects on increasing K^+^ ions concentration on the thermal denaturation of PaBADH followed by ANS fluorescence changes.** Enzyme samples (0.25 mg/ml) in the non-salt buffer (black line), or this buffer plus 37.5 (green line), 100 (blue line) or 250 (red line) mM KCl were heated in the presence of 100 µM ANS in a temperature range of 20–90°C at 1.5°C/min. Fluorescence emission intensities at 482 nm were plotted against temperature. a.u., arbitrary units. The apparent *T*_m_, values determined by following the changes at 222 nm in near-UV CD spectra (shown in Figure 4 of the main article) are indicated by arrows.
(TIF)

Figure S7 **β-Sheets in ALDH enzymes.** (**A**) Surface representation of the SoBADH dimer showing the secondary structure elements in subunit A. α-Helices are shown in red, β-strands in yellow and non-secondary structures in black. (**B**) Surface representation of the SoBADH dimer showing the β-strands of both subunits. The three β-strands of the oligomerization domain of one monomer and the seven β-strands of the catalytic domain of the other monomer form a ten-stranded pleated β-sheet. The figure was generated using PyMOL (DeLano, W. L., 2002. The PyMOL molecular graphics system on World Wide Web http://www.pymol.org/) and the SoBADH crystal coordinates (PDB accession code 4A0M).
(TIF)
