# Supplementary figures and images for: Potassium and Ionic Strength Effects on the Conformational and Thermal Stability of Two Aldehyde Dehydrogenases Reveal Structural and Functional Roles of K+-Binding Sites
Source: PLoS One. 2013 Jan 24;8(1):e54899. doi: 10.1371/journal.pone.0054899 (PMC3554688; doi:10.1371/journal.pone.0054899)

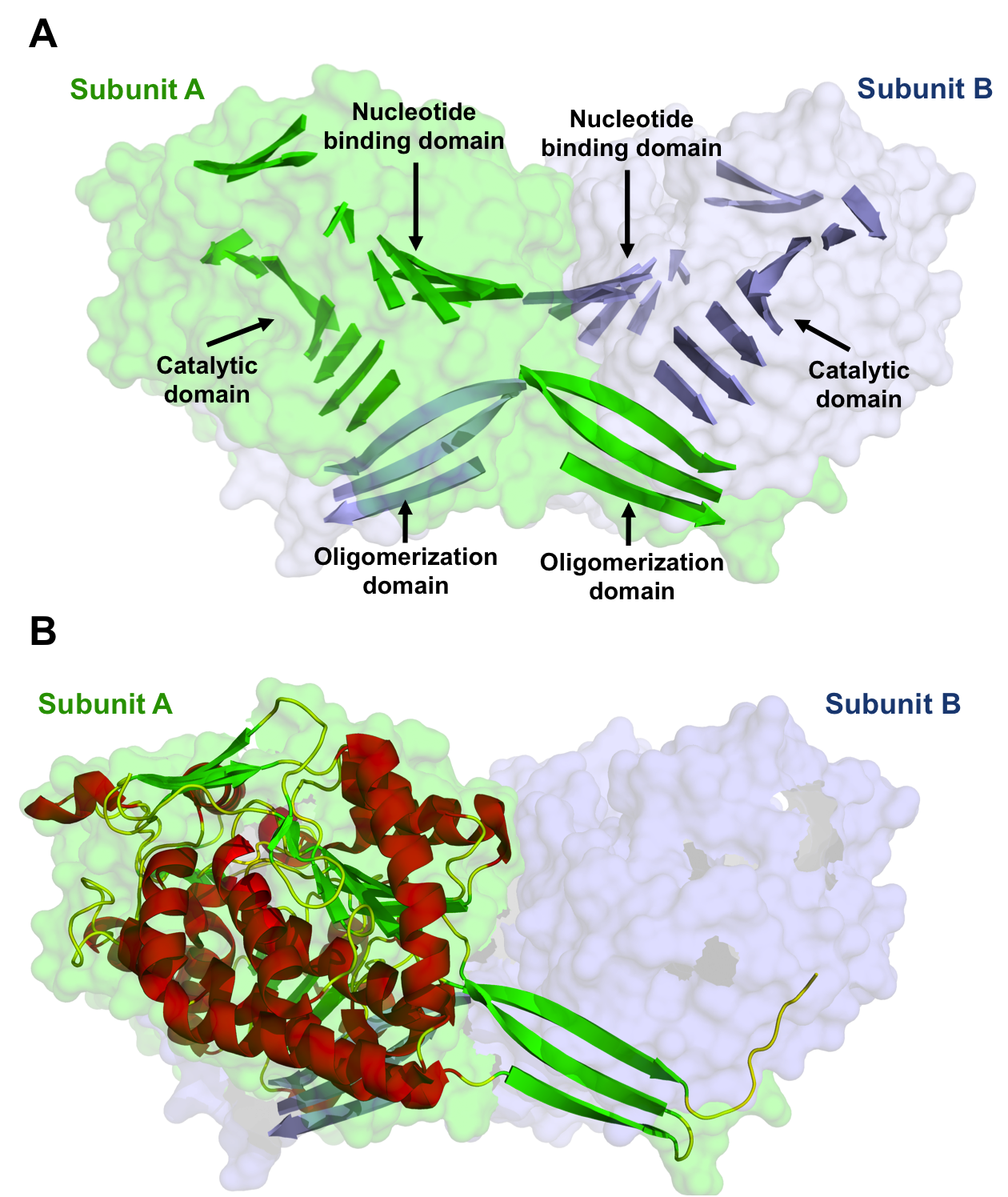

Supplement: Figure S7 — β-Sheets in ALDH enzymes. (A) Surface representation of the SoBADH dimer showing the secondary structure elements in subunit A. α-Helices are shown in red, β-strands in yellow and non-secondary structures in black. (B) Surface representation of the SoBADH dimer showing the β-strands of both subunits. The three β-strands of the oligomerization domain of one monomer and the seven β-strands of the catalytic domain of the other monomer form a ten-stranded pleated β-sheet. The figure was generated using PyMOL (DeLano, W. L., 2002. The PyMOL molecular graphics system on World Wide Web http://www.pymol.org/) and the SoBADH crystal coordinates (PDB accession code 4A0M). (TIF) [file pone.0054899.s007.tif]
